# Supplementary material for: New Kinetic Investigations to Better Understand the Mechanism of Polymorphic Transformations of Pharmaceutical Materials Induced by Milling
Source: Pharmaceutics. 2025 Oct 30;17(11):1404. doi: 10.3390/pharmaceutics17111404 (PMC12655672; doi:10.3390/pharmaceutics17111404)
Supplement: Supplementary file 1 [file pharmaceutics-17-01404-s001.zip › pharmaceutics-3910618-supplementary.pdf]

Table S1. List of pharmaceutical materials exhibiting polymorphic transformation and information relating to these transformations

Pharmaceutical Materials and Molecular Weight	Stating Form and Lattice Parameters (Å)	Final Form and Lattice Parameters (Å)	Relationship	Kinetic Transformation Curve Shape	Information on the Polymorphic Transformation	Tg (°C)	Melting Temperature (°C)	Ref
Sorbitol<br>($C_6H_{14}O_6$)<br>182.17 g/mol	γ<br>a = 24.301<br>b = 20.572<br>c = 4.867<br>α = β = γ = 90°	α<br>a = 9.048<br>b = 4.870<br>c = 18.262<br>α = β = γ = 90°	Monotropic	Sigmoidal	Transformation from the stable γ form to the metastable α form through a transient amorphous phase	−3	95	[1,2]
Bezafibrate<br>($C_{19}H_{20}ClNO_4$)<br>361.82 g/mol	α<br>a = 10.3118<br>b = 17.6601<br>c = 19.7133<br>α = β = γ = 90°	β<br>a= 10.7849<br>b = 15.7886<br>c = 11.4932<br>α = γ = 90°<br>β = 115.875°	Enantiotropic	Sigmoidal	Transformation from the α form (stable at high temperature) to the β form (stable at room temperature) through a transient amorphous phase	40	175	[3]
Sulfamerazine<br>($C_{11}H_{12}N_4O_2S$)<br>264.30 g/mol	I<br>a = 14.474<br>b = 21.953<br>c = 8.203<br>α = β = γ = 90°	II<br>a = 9.145<br>b = 11.704<br>c = 22.884<br>α = β = γ = 90°	Enantiotropic	Sigmoidal	Transformation from form I (stable at high temperature) to a mixture of amorphous phase and form II (stable at room temperature)	62	237	[4], this work
Mannitol<br>($C_6H_{14}O_6$)<br>182.17 g/mol	β<br>a = 5.5381<br>b = 8.580<br>c = 16.795<br>α = β = γ = 90°	α<br>a = 4.8653<br>b = 8.873<br>c = 18.739<br>α = β = γ = 90°	Monotropic	Sigmoidal	Transformation from the stable β form to the metastable α form through a transient amorphous phase	13	166	[5], this work
Glycine<br>($C_2H_5NO_2$)<br>75 g/mol	γ<br>a = b = 7.035<br>c = 5.481<br>α = β = 90°<br>γ = 120°	α<br>a= 5.107<br>b = 12.040<br>c = 5.460<br>α = γ = 90°<br>β = 111.82°	Enantiotropic	Depends on the milling intensity and on the setup<br><br>Sigmoidal for synchrotron experiments	Transformation from the γ form (stable at room temperature) to the α form	Unknown	Unknown	[6,7], this work
Sulfathiazole<br>($C_9H_9N_3O_2S_2$)<br>255.31 g/mol	V<br>a= 10.399<br>b = 15.132<br>c = 14.280<br>α = γ = 90°	I<br>a = 10.554<br>b = 13.220<br>c = 17.050<br>α = γ = 90°	Unknown	Unknown	Transformation from form II, III, IV, and V to a mixture of amorphous phase and form I	67	200	[8,9]

	β = 1.21°<br>IV<br>a = 10.867<br>b = 11.456<br>c = 8.543<br>α = β = 90°<br>γ = 91.87°<br>III<br>a= 17.570<br>b = 8.574<br>c = 15.583<br>α = γ = 90°<br>β = 112.93°<br>II<br>a = 8.235<br>b = 8.550<br>c = 15.558<br>α = γ = 90°<br>β = 93.67°	β = 108.06°<br>I<br>I<br>I						
Ranitidine Hydrochloride $(C_{13}H_{22}N_4O_3S.HCl)$ 350.86 g/mol	I<br>a= 12.1918<br>b = 6.5318<br>c = 22.0382<br>α = γ=90°<br>β = 93.985°	II<br>a = 18.798<br>b = 12.980<br>c = 7.204<br>α = γ = 90°<br>β = 95.09°	Monotropic	Sigmoidal	Transformation from form I (metastable) to form II (stable) through a transient amorphous phase	13–30	I→134–140<br><br>II→140–144	[10,11]
Rivastigmine hydrogen tartrate $(C_{14}H_{22}N_2O_2.C_4H_4O_6)$ 400.42 g/mol	II<br>a = 17.538<br>b =8.326<br>c = 7.261<br>α = γ=90°<br>β = 98.799°	I<br>Unknown	Monotropic	Induction time of 1 h	Transformation from form II (metastable) to form I (stable) through a transient amorphous phase	38.2	II→97.4<br>I→124.5	[12]
Famotidine $(C_8H_{15}N_7O_2S_3)$ 337.44 g/mol	B<br>a = 17.057<br>b =5.335<br>c = 17.776<br>α = γ = 90°<br>β = 116.6°	A<br>a = 11.986<br>b = 7.200<br>c = 16.818<br>α = γ = 90°<br>β = 99.82°	Monotropic	Induction time	Transformation from form B (metastable) to form A (stable) through a transient amorphous phase	50	165	[13,14], this work
Gabapentin $(C_9H_{17}NO_2)$	I<br>a = 14.567	II<br>a = 5.8759	Unknown	Unknown	Transformation from form I to form II	Unknown	166	[15]

171.24 g/mol	b = 9.2153<br>c = 7.6503<br>$\alpha = \gamma = 90°$<br>$\beta = 93.375°$<br>II<br>III	b = 6.9198<br>c = 22.262<br>$\alpha = \gamma = 90°$<br>$\beta = 90.080°$<br>III<br>a = 30.5452<br>b = 5.9268<br>c = 10.8841<br>$\alpha = \gamma = 90°$<br>$\beta = 108.316°$<br>IV<br>a = 14.537<br>b = 6.633<br>c = 9.834<br>$\alpha = \gamma = 90°$<br>$\beta = 105.92°$<br>IV			Transformation from form II to a mixture of form III and IV<br><br>Transformation from form III to form IV			
Indomethacin<br>($C_{19}H_{16}ClNO_4$)<br>357.79 g /mol	γ<br>a = 9.2173<br>b = 9.6060<br>c = 10.8436<br>$\alpha = 69.959$<br>$\beta = 87.1970$<br>$\gamma = 69.501$	α<br>a = 5.4616<br>b =25.310<br>c = 18.152<br>$\alpha = \gamma = 90°$<br>$\beta = 94.38°$	Monotropic	Sigmoidal	Transformation from the γ form (stable) to the α form (metastable) through a transient amorphous phase	47 °C	163	[16–18]
Modafinil<br>($C_{15}H_{15}NO_2S$)<br>273.35 g/mol	I<br>a = 14.5022<br>b = 9.6875<br>c = 20.8445<br>$\alpha = \gamma = 90°$<br>$\beta = 110.17°$<br>IV<br>a = 18.172<br>b = 52.375<br>c = 5.698<br>V<br>Unknown<br>VI<br>Unknown	III<br>a = 14.510<br>b = 9.710<br>c = 19.569<br>$\alpha = \beta = \gamma = 90°$<br>III<br>III<br>III	Monotropic	Unknown	Transformation from form I, IV, V, and VI (metastable) to the III (stable)	43 °C	165	[19]

Fananserin<br>($C_{23}H_{24}FN_3O_2S$)<br>425.52 g/mol	III<br>a = 14.625<br>b =14.370<br>c = 720.356<br>$\alpha = \gamma = 90°$<br>$\beta = 92.84°$<br>IV<br>a = 8.633<br>b =9.714<br>c = 12.270<br>$\alpha = \gamma$=90°<br>$\beta = 96.70°$	I<br>a = 8.359<br>b = 17.228<br>c = 8.089<br>$\alpha = 101.32$<br>$\beta = 110.85$<br>$\gamma = 86.85$<br>I	Monotropic	Induction time of more than 1 h for the IV→I transformation	Transformation from form III (metastable) and IV (stable) to form I (metastable) through a transient amorphous phase	19	III→101<br>IV→99	[20]
Chloramphenicol Palmitate<br>($C_{27}H_{42}Cl_2N_2O_6$)<br>561.54 g/mol	C<br>Unknown<br>B<br>Unknown	B<br>Unknown<br>A<br>a = 7.805<br>b = 52.503<br>c = 7.414<br>$\alpha = \beta = \gamma = 90°$	Enantiotropic<br><br>Monotropic	Exponential Relaxation<br><br>Sigmoidal	Transformation from form C (metastable) to form B (metastable) then to form A (stable)	Unknown	C→B à 64.5<br>A→90.3<br>B→86.7	[21]
Cimetidine<br>($C_{10}H_{16}N_6S$)<br>252.34 g/mol	B<br>a = 55.45<br>b =5<br>c = 18.72<br>$\alpha = \gamma$=90°<br>$\beta = 100.4°$<br>C<br>a = 82.904<br>b =4.85<br>c = 18.760<br>$\alpha = \gamma$=90°<br>$\beta = 74.34°$	A<br>a = 10.7029<br>b =18.8262<br>c = 6.8266<br>$\alpha = \gamma$=90°<br>$\beta = 111.306°$<br>A	Monotropic	Unknown	Transformation from form B and C (both metastable) to form A (stable) through a transient amorphous phase	43	A→140–152<br>B→142–145<br>C→145–154	[22,23]
Phenylbutazone<br>($C_{19}H_{20}N_2O_2$)<br>308.37 g/mol	4 °C Milling<br>α<br>a = 21.415<br>b =5.7295<br>c =27.782<br>$\alpha = \gamma$=90°<br>$\beta = 108.4°$<br>ß	4 °C Milling<br>ε<br>Unknown<br>ε<br>ε<br>35 °C Milling<br>δ<br>δ	ß/δ Monotropic<br><br>α/ß Enantiotropic<br><br>α/δ Enantiotropic	Unknown	At 4 °C: transformation from the α, β, and δ form to the ε form after several hours of milling<br>At 35 °C: transformation from the α and β form to the δ form after several hours of milling	4	α→91.2<br>ß→93.3<br>δ→101.4	[24–26]

	Unknown<br>δ<br>Unknown<br>35 °C Miling<br>α<br>ß<br>δ	δ						
Nolomirole Hydrochlorride<br>($C_{19}H_{28}ClNO_4$)<br>369.88 g/mol	α<br>Unknown	β<br>Unknown	Unknown	Unknown	Transformation from the α form to the β form	Unknown	Unknown	[27]
Caffeine<br>($C_8H_{10}N_4O_2$)<br>194.19 g/mol	I<br>a = 14.9372<br>b = 14.9372<br>c = 6.8980<br>α= β =90<br>γ = 120<br>II	II<br>a = 43.0390<br>b =15.06758<br>c = 6.95314<br>α = γ=90°<br>β = 99.0274°<br>I	Enantiotropic	Transformation too fast to observe kinetics	Transformation from form I (metastable) to form II (stable)<br>Transformation from form II (stable) to form I (metastable)	−17	227	[28–30]

References

1. Dupont, A. *et al.* Kinetics and mechanism of polymorphic transformation of sorbitol under mechanical milling. *Int. J. Pharm.* **590**, 119902 (2020).10
2. Dupont, A., Guerain, M., Danède, F. & Willart, J. F. Evidence of transient amorphization during the polymorphic transformation of sorbitol induced by milling. *Int. J. Pharm.* **623**, 121929 (2022).14
3. Dudognon, E., Danède, F. & Guerain, M. Milling-Induced Phase Transformations, Underlying Mechanisms, and Resulting Physical States in an Enantiotropic System: The Case of Bezafibrate. *Cryst. Growth Des.* **22**, 363–378 (2022).11
4. Macfhionnghaile, P. *et al.* Effects of Ball-Milling and Cryomilling on Sulfamerazine Polymorphs: A Quantitative Study. *J. Pharm. Sci.* **103**, 1766–1778 (2014).17
5. Martinetto, P. *et al.* Structural Transformations of d-Mannitol Induced by in Situ Milling Using Real Time Powder Synchrotron Radiation Diffraction. *Cryst. Growth Des.* **17**, 6111–6122 (2017).12
6. Matsuoka, M., Hirata, J. & Yoshizawa, S. Kinetics of solid-state polymorphic transition of glycine in mechano-chemical processing. *Chem. Eng. Res. Des.* **88**, 1169–1173 (2010).15
7. Dupont, A. Cinétiques de transformations de produits pharmaceutiques sous broyage. (2022).26
8. Hu, Y. *et al.* Solid-State Transformations of Sulfathiazole Polymorphs: The Effects of Milling and Humidity. *Cryst. Growth Des.* **13**, 3404–3413 (2013).30
9. Aji, D. P. B., Khouri, J. & Johari, G. P. Non-exponential relaxation, fictive temperatures, and dispersive kinetics in the liquid-glass-liquid transition range of acetaminophen, sulfathiazole, and their mixtures. *J. Chem. Phys.* **141**, 174507 (2014).
10. Madan, T. & Kakkar, A. Preparation and Characterization of Ranitidine-HC1 Crystals. *Drug Dev. Ind. Pharm.* **20**, 1571–1588 (1994).
11. Chieng, N., Zujovic, Z., Bowmaker, G., Rades, T. & Saville, D. Effect of milling conditions on the solid-state conversion of ranitidine hydrochloride form 1. *Int. J. Pharm.* **327**, 36–44 (2006). 33
12. Amaro, M. I., Simon, A., Cabral, L. M., de Sousa, V. P. & Healy, A. M. Rivastigmine hydrogen tartrate polymorphs: Solid-state characterisation of transition and polymorphic conversion via milling. *Solid State Sci.* **49**, 29–36 (2015). 34
13. Lin, S.-Y., Cheng, W.-T. & Wang, S.-L. Thermodynamic and kinetic characterization of polymorphic transformation of famotidine during grinding. *Int. J. Pharm.* **318**, 86–91 (2006). 16
14. Mahlin, D. & Bergström, C. A. S. Early drug development predictions of glass-forming ability and physical stability of drugs. *Eur. J. Pharm. Sci.* **49**, 323–332 (2013).
15. Lin, S.-Y., Hsu, C.-H. & Ke, W.-T. Solid-state transformation of different gabapentin polymorphs upon milling and co-milling. *Int. J. Pharm.* **396**, 83–90 (2010).
16. Otsuka, M., Otsuka, K. & Kaneniwa, N. Relation Between Polymorphic Transformation Pathway During Grinding and the Physicochemical Properties of Bulk Powders for Pharmaceutical Preparations. *Drug Dev. Ind. Pharm.* **20**, 1649–1660 (1994).
17. Desprez, S. Transformation de phases induites par broyage dans un composé moléculaire : l'indométhacine. (2004).31
18. Luisi, B. S., Medek, A., Liu, Z., Mudunuri, P. & Moulton, B. Milling-Induced Disorder of Pharmaceuticals: One-Phase or Two-Phase System? *J. Pharm. Sci.* **101**, 1475–1485 (2012).
19. Linol, J., Morelli, T., Petit, M. N. & Coquerel, G. Inversion of the relative stability between two polymorphic forms of (±) modafinil under dry high-energy milling: Comparisons with results obtained under wet high-energy milling. in *Crystal Growth and Design* vol. 7 1608–1611 (American Chemical Society , 2007).
20. Gusseme, A. De, Neves, C., Willart, J. F., Rameau, A. & Descamps, M. Ordering and disordering of molecular solids upon mechanical milling: the case of fananserine. *J. Pharm. Sci.* **97 11**, 5000–5012 (2008).8
21. Otsuka, M. & Kaneniwa, N. Effect of seed crystals on solid-state transformation of polymorphs of chloramphenicol palmitate during grinding1. *J. Pharm. Sci.* **75**, 506–511 (1986).
22. Thayyil, M. S. Fragility of Cimetidine Drug Probed by Broadband Dielectric Spectroscopy.

Transl. Med. **04**, (2014).

23. Bauer-Brandl, A. Polymorphic transitions of cimetidine during manufacture of solid dosage forms. *Int. J. Pharm.* **140**, 195–206 (1996).32
24. KANENIWA, N., ICHIKAWA, J.-I. & MATSUMOTO, T. Preparation of Phenylbutazone Polymorphs and Their Transformation in Solution. *Chem. Pharm. Bull. (Tokyo).* **36**, 1063–1073 (1988).
25. FUKUOKA, E., MAKITA, M. & YAMAMURA, S. Glassy State of Pharmaceuticals. III. : Thermal Properties and Stability of Glassy Pharmaceuticals and Their Binary Glass Systems. *Chem. Pharm. Bull. (Tokyo).* **37**, 1047–1050 (1989).
26. Kohno, Y. *et al.* Molecular Dynamics Studies of the Structural Change in 1,3-Diamino-2,4,6-trinitrobenzene (DATB) in the Crystalline State under High Pressure. *J. Phys. Chem. A* **113**, 2551–2560 (2009).
27. Taddei, P., Torreggiani, A. & Fini, G. Vibrational study of polymorphism of tetralin derivative for treatment of cardiovascular diseases. *Biopolymers* **67**, 289–293 (2002).
28. Pirttimäki, J., Laine, E., Ketolainen, J. & Paronen, P. Effects of grinding and compression on crystal structure of anhydrous caffeine. *Int. J. Pharm.* **95**, 93–99 (1993).
29. Descamps, M., Correia, N. T., Derollez, P., Danede, F. & Capet, F. Plastic and Glassy Crystal States of Caffeine. *J. Phys. Chem. B* **109**, 16092–16098 (2005).
30. Descamps, M. & Decroix, A. A. Polymorphism and disorder in caffeine: Dielectric investigation of molecular mobilities. *J. Mol. Struct.* **1078**, 165–173 (2014).
